# On Muriat and Tartrit of Antimony

**Published:** 1805-05-01

**Authors:** Robert Stott

**Affiliations:** Surgeon. Dumfries


### *On Muriat and Tartrit of Antimony: communicated by* Mr. STOTT, *Surgeon, of Dumfries.*

[ Continued from Vol. XI. pp. 497—505. ]

I Shall now give an account of some trials I made, with the view of improving the methods of preparing muriat of antimony, and which the great importance of the subject induced me to make. After reading Mr. Scheele's valuable essay on manganese, and observing that in a mixture of manganese, muriat of soda, diluted sulphuric acid and gold, it was found that gold was taken up in solution, I never doubted of success in distilling muriat of antimony in the same way. More confident of success than I ought to have been, I put into a large retort two pounds of muriat of soda, two pounds of black manganese, one pound two ounces of sulphurated antimony, and poured upon them one pound of sulphuric acid previously diluted in one pound and a half of water. The whole being well agitated, and the vessel set on warm sand, an effervescence commenced, with a strong suffocating smell of oxygenated muriatic acid. The retort was placed for distillation, and a receiver applied, luted with flour paste. From mid day the distillation was continued till nine o'clock in the afternoon, and during most of that time an effervescence went on with the oppressive smell of oxygenated muriatic acid, which penetrated through the lute. About that time the mixture was distilled to dryness, and the retort cracked; and on lifting it out of the furnace, I run considerable risk, as the fumes issued out very copiously, and were intolerable to the lungs. Two pounds of fluid had distilled over into the receiver, of a light yellow or greenish tinge, and smelt strongly of oxygenated muriatic acid. It effervesced strongly with alkalies, fixed and volatile, but which, however, precipitated nothing from it; of course, not a particle of oxydised antimony had been carried up by it.

Next day I set about examining the residue of my experiment, periment, and found a hard solid mass, of a grey colour, full of little crystals, and so firm that it required repeated strokes of a hammer to break it. Three ounces of this mass was pounded and put into a mattrass, one pound of water added, and a digesting heat applied. In order to be certain whether any of the antimony were so much oxydized as to constitute muriat of antimony, I put two ounces of muriat of soda into this solution, and one ounce of sulphuric acid diluted, and continued the digestion for several hours. The solution, which was now yellow, was filtered through paper into a jug, and largely diluted with boiling water; but still not a particle of pulvis algerothi was precipitated. Alkali of potash was next added, when a brisk effervescence was excited; but after this ceased, on continuing to add, a white precipitate was thrown down, which seemed to agree with what Mr. Scheele calls phlogisticated manganese, as it turned black when roasted with a red heat on an iron plate. The insoluble matter on the bottom of the mattras was partly antimony, little or nothing changed, and partly a grey powder.

In order, if possible, to obtain muriat of antimony by manganese and muriatic acid, I took one ounce of the mass first above mentioned, one ounce of powdered manganese, and one ounce of muriat of soda, and set them to digest with half an ounce of sulphuric acid. Having digested this mixture during three days, I saw no more appearance of solution than at first, as it had the same black colour; and allowed to settle, the solution was yellowish, and not milky, like muriat of antimony viâ humidâ. I therefore boiled the mixture to dryness, and towards the end, when the last of muriatic acid went off, a little whitish substance covered the inside of the upper part of the vessel, and seemed to be a little phlogisticated manganese carried up by the muriatic acid; it was in very small quantity.

In my next essay I mixed two ounces of sulphurated antimony and four ounces of sulphuric acid, and boiled to dryness, and at last gave a pretty strong heat till yellow sulphur appeared at the mouth of the vessel. The mass was now of a dirty white colour, and somewhat black at top. The vapours were exceedingly offensive. The sulphat of antimony weighed about two ounces and a half. It was quite dry and friable. It was pounded and mixed with two ounces of muriat of soda and put into a retort. Heat was next applied to this mass, which looked blue like quicksilver, and its corrosive muriat rubbed together with the view of distilling muriat of antimony from it. At first a dewy a dewy vapour rather whitish rose, and then a milky substance distilled into the receiver, and some of it lay along the under side of the stem of the retort, but afterwards an ochry matter rose and dirtied the white matter, and then a clear liquor dropped into the receiver. What remained on the neck of the retort was soft, but did not liquefy by heat; and when scraped out, and water dropped on it, looked like a paste of chalk and water. The clear fluid in the receiver poured off from the white matter, and boiling water added to it, became milky, or as it were curdled; but diluting largely, the whole fluid and soft matter grew a little reddish like Kermes mineral. Having washed and dried the precipitate and other matter distilled over, it weighed one drachm and two scruples. Again I deflagrated four ounces of sulphurated antimony with six ounces of nitrat of potash in a hot crucible, and when cold pounded it. Now it weighed seven ounces. It was rubbed with four ounces of sulphat of iron and four ounces of muriat of soda into an ochry coloured mass. I put it into a retort and distilled off about three ounces of a clear fluid of a pungent disagreeable smell. An ochry matter had also come over and covered about half of the inside of the receiver, and stuck to it so as not to be washed off with water. The fluid I found to be mere phlegm void of taste, and giving no precipitate with water or alkalies. The retort was found cracked into many pieces, and the residue in it was now partly a fused salt, very hard as if vitrified, and under that there seemed to be fused antimony little changed. With great difficulty I pounded the whole, as it was very hard. It was of a dirty blue colour; I put it into a digester, washed it two or three times with hot water, and then added two ounces of muriat of soda and two ounces of diluted sulphuric acid, and exposed this mixture to digestion. On diluting with hot water, however, a slight milkiness was produced, but scarcely any sensible quantity of pulvis algerothi was precipitated.

The conclusion I deduce from these three experiments, which had analogy in their favour, and might have been supported by strong and plausible arguments before trial, is, that the intelligent chemist ought never to trust to reasoning alone in the business of experimental philosophy. "Hypotheses enim," says Sir Isaac Newton, "in philosophia quæ circa experimenta versatur pro nihilo sunt habendæ.

I am, &c.

ROBERT STOTT, Surgeon.

*Dumfries, March 27, 1805.*

